# Hexaaqua­cadmium(II) bis­[4-(2-hydroxy­benzyl­ideneamino)benzene­sulfonate] dihydrate

**DOI:** 10.1107/S1600536808010878

**Published:** 2008-04-23

**Authors:** Xi-Shi Tai, Jun Xu, Yi-Min Feng, Zu-Pei Liang

**Affiliations:** a Department of Chemistry and Chemical Engineering, Weifang University, Weifang 261061, People’s Republic of China; b Weifang Institute of Supervision and Inspection of Product Quality, Weifang 261031, People’s Republic of China

## Abstract

In the title compound, [Cd(H_2_O)_6_](C_13_H_10_NO_4_S)_2_·2H_2_O, the Cd atom (site symmetry 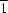) adopts a regular octa­hedral coordination and the anion is stabilized by an intra­molecular O—H⋯N hydrogen bond. O—H⋯O hydrogen bonds involving the coordinated and uncoordinated water mol­ecules lead to a three-dimensional network.

## Related literature

For related literature, see: Tai *et al.* (2008 ▶).
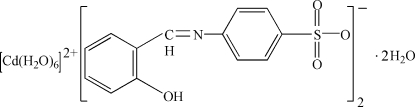


## Experimental

### 

#### Crystal data

[Cd(H_2_O)_6_](C_13_H_10_NO_4_S)_2_·2H_2_O
*M* *_r_* = 809.09
Monoclinic, 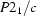

*a* = 18.464 (2) Å
*b* = 6.1488 (8) Å
*c* = 14.5701 (12) Å
β = 92.226 (2)°
*V* = 1652.9 (3) Å^3^
*Z* = 2
Mo *K*α radiation
μ = 0.86 mm^−1^
*T* = 298 (2) K
0.48 × 0.45 × 0.18 mm

#### Data collection

Bruker SMART CCD diffractometer
Absorption correction: multi-scan (*SADABS*; Bruker, 2000 ▶) *T*_min_ = 0.682, *T*_max_ = 0.860
7936 measured reflections
2904 independent reflections
2447 reflections with *I* > 2σ(*I*)
*R*_int_ = 0.035

#### Refinement

*R*[*F* ^2^ > 2σ(*F* ^2^)] = 0.028
*wR*(*F* ^2^) = 0.075
*S* = 1.06
2904 reflections
214 parameters
H-atom parameters constrained
Δρ_max_ = 0.28 e Å^−3^
Δρ_min_ = −0.60 e Å^−3^

### 

Data collection: *SMART* (Bruker, 2000 ▶); cell refinement: *SAINT* (Bruker, 2000 ▶); data reduction: *SAINT*; program(s) used to solve structure: *SHELXS97* (Sheldrick, 2008 ▶); program(s) used to refine structure: *SHELXL97* (Sheldrick, 2008 ▶); molecular graphics: *SHELXTL* (Sheldrick, 2008 ▶); software used to prepare material for publication: *SHELXTL*.

## Supplementary Material

Crystal structure: contains datablocks global, I. DOI: 10.1107/S1600536808010878/hb2722sup1.cif

Structure factors: contains datablocks I. DOI: 10.1107/S1600536808010878/hb2722Isup2.hkl

Additional supplementary materials:  crystallographic information; 3D view; checkCIF report

## Figures and Tables

**Table 1:** Selected bond lengths (Å)

Cd1—O5	2.2684 (19)
Cd1—O7	2.2826 (18)
Cd1—O6	2.2862 (17)

**Table 2:** Hydrogen-bond geometry (Å, °)

*D*—H⋯*A*	*D*—H	H⋯*A*	*D*⋯*A*	*D*—H⋯*A*
O4—H4⋯N1	0.82	1.89	2.611 (3)	147
O5—H5*A*⋯O1^i^	0.85	2.07	2.909 (3)	170
O5—H5*B*⋯O8^ii^	0.85	1.92	2.750 (3)	167
O6—H6*A*⋯O8^iii^	0.85	1.93	2.778 (3)	177
O6—H6*B*⋯O2^iv^	0.85	1.97	2.802 (3)	166
O7—H7*A*⋯O1	0.85	1.95	2.793 (3)	174
O7—H7*B*⋯O3^i^	0.85	1.91	2.757 (3)	172
O8—H8*A*⋯O2	0.85	1.90	2.750 (3)	176
O8—H8*B*⋯O3^i^	0.85	2.00	2.851 (3)	176

Symmetry codes: (i) 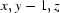; (ii) 
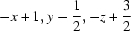
; (iii) 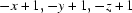; (iv) 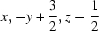.

**Fig. 1.**
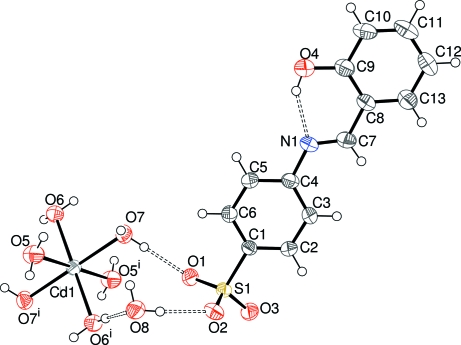
The molecular structure of (I) showing 50% displacement ellipsoids (arbitrary spheres for the H atoms). The hydrogen bonds are indicated by double-dashed lines. Symmetry code: (i) 1-x, 1-y, 1-z.

## supplementary crystallographic information

### Comment

As part of our ongoing studies of the synthesis and coordination chemistry of Schiff-base ligands (e.g. Tai *et al.*, 2008), we now report the synthesis and structure of the title compound, (I), (Fig. 1), in which the organic species does not coordiate to the metal and a hydrated molecular salt arises.

The Cd atom (site symmetry 1) in (I) is bonded to six water molecules (Table 1).  The anion is stablisied by an intramolecular O-H···N hydrogen bond (Table 2), which perhaps correlates with the fact that the aromatic rings are almost co-planar [dihedral angle = 4.09 (14)°].

The water molecules, both bound and unbound, participate in O-H···O hydrogen bonds to link the component speices into a three-dimensional network.

### Experimental

1 mmol of cadmium nitrate was added to a solution of salicylaldehyde-4-aminobenzene sulfonic acid (1 mmol) in 10 ml of 95% ethanol. The mixture was stirred for 2 h at refluxing temperature. Evaporating some ethanol, clear blocks of (I) were obtained after one weeks.

### Refinement

The H atoms were placed geometrically (C—H = 0.93–0.96 Å, O—H = 0.85Å) and refined as riding with *U*_iso_(H) = 1.2*U*_eq_(carrier).

### Crystal data

**Table:**

[Cd(H_2_O)_6_](C_13_H_10_NO_4_S)_2_·2H_2_O	*F*_000_ = 828
*M**_r_* = 809.09	*D*_x_ = 1.626 Mg m^−^^3^
Monoclinic, *P*2_1_/*c*	Mo *K*α radiation λ = 0.71073 Å
Hall symbol: -P 2ybc	Cell parameters from 4564 reflections
*a* = 18.464 (2) Å	θ = 2.6–27.9º
*b* = 6.1488 (8) Å	µ = 0.86 mm^−^^1^
*c* = 14.5701 (12) Å	*T* = 298 (2) K
β = 92.226 (2)º	Block, colourless
*V* = 1652.9 (3) Å^3^	0.48 × 0.45 × 0.18 mm
*Z* = 2	

### Data collection

**Table:**

Bruker SMART CCD diffractometer	2904 independent reflections
Radiation source: fine-focus sealed tube	2447 reflections with *I* > 2σ(*I*)
Monochromator: graphite	*R*_int_ = 0.035
*T* = 298(2) K	θ_max_ = 25.0º
ω scans	θ_min_ = 2.2º
Absorption correction: multi-scan(SADABS; Bruker, 2000)	*h* = −21→21
*T*_min_ = 0.682, *T*_max_ = 0.860	*k* = −7→5
7936 measured reflections	*l* = −17→16

### Refinement

**Table:**

Refinement on *F*^2^	Secondary atom site location: difference Fourier map
Least-squares matrix: full	Hydrogen site location: inferred from neighbouring sites
*R*[*F*^2^ > 2σ(*F*^2^)] = 0.028	H-atom parameters constrained
*wR*(*F*^2^) = 0.075	*w* = 1/[σ^2^(*F*_o_^2^) + (0.0376*P*)^2^ + 0.6281*P*] where *P* = (*F*_o_^2^ + 2*F*_c_^2^)/3
*S* = 1.06	(Δ/σ)_max_ < 0.001
2904 reflections	Δρ_max_ = 0.28 e Å^−^^3^
214 parameters	Δρ_min_ = −0.60 e Å^−^^3^
Primary atom site location: structure-invariant direct methods	Extinction correction: none

### Special details

**Table:**

Geometry. All e.s.d.'s (except the e.s.d. in the dihedral angle between two l.s. planes) are estimated using the full covariance matrix. The cell e.s.d.'s are taken into account individually in the estimation of e.s.d.'s in distances, angles and torsion angles; correlations between e.s.d.'s in cell parameters are only used when they are defined by crystal symmetry. An approximate (isotropic) treatment of cell e.s.d.'s is used for estimating e.s.d.'s involving l.s. planes.
Refinement. Refinement of *F*^2^ against ALL reflections. The weighted *R*-factor *wR* and goodness of fit *S* are based on *F*^2^, conventional *R*-factors *R* are based on *F*, with *F* set to zero for negative *F*^2^. The threshold expression of *F*^2^ > σ(*F*^2^) is used only for calculating *R*-factors(gt) *etc*. and is not relevant to the choice of reflections for refinement. *R*-factors based on *F*^2^ are statistically about twice as large as those based on *F*, and *R*- factors based on ALL data will be even larger.

### Fractional atomic coordinates and isotropic or equivalent isotropic displacement parameters (Å^2^)

**Table:**

	*x*	*y*	*z*	*U*_iso_*/*U*_eq_	
Cd1	0.5000	0.5000	0.5000	0.03115 (11)	
N1	0.00853 (11)	0.8935 (4)	0.61988 (16)	0.0392 (5)	
O1	0.35962 (10)	0.9727 (3)	0.59697 (14)	0.0419 (5)	
O2	0.34686 (10)	0.9949 (3)	0.76142 (14)	0.0424 (5)	
O3	0.33264 (10)	1.3176 (3)	0.66883 (14)	0.0425 (5)	
O4	−0.09042 (11)	0.6152 (4)	0.56383 (17)	0.0655 (7)	
H4	−0.0493	0.6618	0.5742	0.098*	
O5	0.50034 (11)	0.1696 (3)	0.56929 (14)	0.0472 (5)	
H5A	0.4573	0.1274	0.5779	0.057*	
H5B	0.5235	0.1750	0.6208	0.057*	
O6	0.46644 (10)	0.3400 (3)	0.36324 (12)	0.0425 (5)	
H6A	0.4997	0.3570	0.3251	0.051*	
H6B	0.4279	0.3987	0.3412	0.051*	
O7	0.38005 (10)	0.5588 (3)	0.52336 (13)	0.0415 (5)	
H7A	0.3745	0.6886	0.5418	0.050*	
H7B	0.3663	0.4736	0.5652	0.050*	
O8	0.42645 (10)	0.6168 (3)	0.76420 (13)	0.0462 (5)	
H8A	0.4035	0.7366	0.7646	0.055*	
H8B	0.4000	0.5230	0.7362	0.055*	
S1	0.32404 (3)	1.08214 (11)	0.67158 (5)	0.03098 (16)	
C1	0.23015 (14)	1.0294 (4)	0.65692 (18)	0.0296 (6)	
C2	0.18127 (14)	1.1822 (5)	0.6840 (2)	0.0463 (8)	
H2	0.1976	1.3125	0.7098	0.056*	
C3	0.10786 (15)	1.1428 (5)	0.6730 (2)	0.0523 (9)	
H3	0.0749	1.2470	0.6912	0.063*	
C4	0.08304 (14)	0.9485 (5)	0.63495 (19)	0.0343 (6)	
C5	0.13250 (15)	0.7954 (5)	0.6086 (2)	0.0447 (7)	
H5	0.1163	0.6647	0.5830	0.054*	
C6	0.20627 (14)	0.8343 (5)	0.6199 (2)	0.0440 (7)	
H6	0.2395	0.7296	0.6027	0.053*	
C7	−0.04225 (15)	1.0304 (5)	0.6320 (2)	0.0406 (7)	
H7	−0.0302	1.1709	0.6507	0.049*	
C8	−0.11807 (14)	0.9738 (4)	0.61762 (19)	0.0368 (7)	
C9	−0.13909 (15)	0.7700 (5)	0.5839 (2)	0.0437 (7)	
C10	−0.21291 (15)	0.7219 (5)	0.5712 (2)	0.0522 (8)	
H10	−0.2273	0.5861	0.5491	0.063*	
C11	−0.26382 (16)	0.8743 (6)	0.5911 (2)	0.0521 (8)	
H11	−0.3127	0.8403	0.5826	0.063*	
C12	−0.24418 (16)	1.0779 (6)	0.6235 (2)	0.0529 (8)	
H12	−0.2793	1.1808	0.6361	0.064*	
C13	−0.17160 (15)	1.1260 (5)	0.6369 (2)	0.0464 (7)	
H13	−0.1580	1.2623	0.6592	0.056*	

### Atomic displacement parameters (Å^2^)

**Table:**

	*U*^11^	*U*^22^	*U*^33^	*U*^12^	*U*^13^	*U*^23^
Cd1	0.03180 (16)	0.03028 (17)	0.03128 (16)	0.00014 (11)	0.00026 (11)	−0.00045 (11)
N1	0.0266 (12)	0.0439 (14)	0.0467 (14)	−0.0041 (11)	−0.0031 (10)	−0.0035 (11)
O1	0.0316 (10)	0.0456 (12)	0.0490 (12)	−0.0008 (8)	0.0082 (9)	−0.0114 (9)
O2	0.0331 (10)	0.0496 (13)	0.0438 (12)	−0.0010 (8)	−0.0079 (9)	0.0034 (9)
O3	0.0378 (10)	0.0300 (10)	0.0600 (13)	−0.0074 (8)	0.0050 (9)	−0.0035 (9)
O4	0.0424 (12)	0.0513 (14)	0.102 (2)	−0.0018 (11)	−0.0095 (12)	−0.0244 (14)
O5	0.0534 (12)	0.0412 (12)	0.0463 (12)	−0.0071 (10)	−0.0058 (9)	0.0082 (9)
O6	0.0426 (11)	0.0443 (11)	0.0399 (11)	0.0034 (9)	−0.0062 (9)	−0.0043 (9)
O7	0.0358 (10)	0.0384 (11)	0.0508 (12)	0.0016 (9)	0.0067 (9)	−0.0007 (9)
O8	0.0505 (12)	0.0381 (12)	0.0492 (12)	0.0031 (9)	−0.0094 (9)	−0.0032 (9)
S1	0.0249 (3)	0.0302 (3)	0.0378 (4)	−0.0031 (3)	0.0007 (3)	−0.0016 (3)
C1	0.0258 (13)	0.0322 (15)	0.0306 (13)	−0.0012 (11)	0.0004 (10)	−0.0005 (11)
C2	0.0330 (15)	0.0418 (17)	0.064 (2)	−0.0024 (13)	−0.0032 (14)	−0.0210 (15)
C3	0.0283 (14)	0.052 (2)	0.076 (2)	0.0050 (14)	−0.0011 (14)	−0.0259 (17)
C4	0.0285 (14)	0.0411 (16)	0.0332 (14)	−0.0034 (12)	−0.0013 (11)	−0.0016 (12)
C5	0.0329 (14)	0.0346 (16)	0.066 (2)	−0.0045 (13)	−0.0012 (13)	−0.0125 (14)
C6	0.0294 (14)	0.0326 (16)	0.070 (2)	−0.0018 (12)	0.0036 (13)	−0.0113 (14)
C7	0.0344 (15)	0.0415 (17)	0.0458 (17)	−0.0083 (13)	0.0000 (13)	0.0011 (13)
C8	0.0274 (14)	0.0446 (18)	0.0384 (15)	−0.0035 (12)	−0.0001 (11)	0.0035 (12)
C9	0.0354 (15)	0.0475 (18)	0.0477 (17)	−0.0032 (14)	−0.0036 (13)	0.0003 (14)
C10	0.0398 (17)	0.055 (2)	0.061 (2)	−0.0140 (15)	−0.0127 (14)	0.0020 (16)
C11	0.0293 (15)	0.076 (3)	0.0501 (19)	−0.0080 (16)	−0.0062 (13)	0.0125 (17)
C12	0.0331 (16)	0.069 (2)	0.057 (2)	0.0102 (16)	0.0052 (14)	0.0098 (17)
C13	0.0389 (16)	0.0487 (19)	0.0517 (18)	0.0001 (14)	0.0025 (13)	0.0026 (14)

### Geometric parameters (Å, °)

**Table:**

Cd1—O5	2.2684 (19)	C1—C2	1.371 (4)
Cd1—O5^i^	2.2684 (19)	C1—C6	1.380 (4)
Cd1—O7	2.2826 (18)	C2—C3	1.380 (4)
Cd1—O7^i^	2.2826 (18)	C2—H2	0.9300
Cd1—O6^i^	2.2862 (17)	C3—C4	1.388 (4)
Cd1—O6	2.2862 (17)	C3—H3	0.9300
N1—C7	1.278 (4)	C4—C5	1.377 (4)
N1—C4	1.425 (3)	C5—C6	1.386 (4)
O1—S1	1.4565 (19)	C5—H5	0.9300
O2—S1	1.462 (2)	C6—H6	0.9300
O3—S1	1.4570 (19)	C7—C8	1.450 (4)
O4—C9	1.349 (4)	C7—H7	0.9300
O4—H4	0.8200	C8—C9	1.395 (4)
O5—H5A	0.8499	C8—C13	1.398 (4)
O5—H5B	0.8501	C9—C10	1.400 (4)
O6—H6A	0.8500	C10—C11	1.366 (4)
O6—H6B	0.8501	C10—H10	0.9300
O7—H7A	0.8500	C11—C12	1.381 (5)
O7—H7B	0.8500	C11—H11	0.9300
O8—H8A	0.8499	C12—C13	1.379 (4)
O8—H8B	0.8499	C12—H12	0.9300
S1—C1	1.768 (3)	C13—H13	0.9300
			
O5—Cd1—O5^i^	180.0	C1—C2—C3	120.1 (3)
O5—Cd1—O7	93.52 (7)	C1—C2—H2	120.0
O5^i^—Cd1—O7	86.48 (7)	C3—C2—H2	120.0
O5—Cd1—O7^i^	86.48 (7)	C2—C3—C4	120.3 (3)
O5^i^—Cd1—O7^i^	93.52 (7)	C2—C3—H3	119.8
O7—Cd1—O7^i^	180.0	C4—C3—H3	119.8
O5—Cd1—O6^i^	90.09 (7)	C5—C4—C3	119.2 (2)
O5^i^—Cd1—O6^i^	89.91 (7)	C5—C4—N1	116.2 (2)
O7—Cd1—O6^i^	91.93 (7)	C3—C4—N1	124.6 (3)
O7^i^—Cd1—O6^i^	88.07 (7)	C4—C5—C6	120.5 (3)
O5—Cd1—O6	89.91 (7)	C4—C5—H5	119.7
O5^i^—Cd1—O6	90.09 (7)	C6—C5—H5	119.7
O7—Cd1—O6	88.07 (7)	C1—C6—C5	119.6 (3)
O7^i^—Cd1—O6	91.93 (7)	C1—C6—H6	120.2
O6^i^—Cd1—O6	180.0	C5—C6—H6	120.2
C7—N1—C4	122.1 (2)	N1—C7—C8	122.1 (3)
C9—O4—H4	109.5	N1—C7—H7	119.0
Cd1—O5—H5A	110.6	C8—C7—H7	119.0
Cd1—O5—H5B	110.5	C9—C8—C13	118.9 (3)
H5A—O5—H5B	108.8	C9—C8—C7	121.4 (3)
Cd1—O6—H6A	109.8	C13—C8—C7	119.7 (3)
Cd1—O6—H6B	110.0	O4—C9—C8	122.1 (2)
H6A—O6—H6B	108.4	O4—C9—C10	118.4 (3)
Cd1—O7—H7A	109.0	C8—C9—C10	119.5 (3)
Cd1—O7—H7B	109.2	C11—C10—C9	120.1 (3)
H7A—O7—H7B	107.9	C11—C10—H10	120.0
H8A—O8—H8B	108.3	C9—C10—H10	120.0
O1—S1—O3	112.68 (12)	C10—C11—C12	121.4 (3)
O1—S1—O2	112.09 (12)	C10—C11—H11	119.3
O3—S1—O2	111.19 (12)	C12—C11—H11	119.3
O1—S1—C1	107.08 (12)	C13—C12—C11	118.9 (3)
O3—S1—C1	106.63 (11)	C13—C12—H12	120.5
O2—S1—C1	106.74 (12)	C11—C12—H12	120.5
C2—C1—C6	120.2 (2)	C12—C13—C8	121.2 (3)
C2—C1—S1	119.5 (2)	C12—C13—H13	119.4
C6—C1—S1	120.2 (2)	C8—C13—H13	119.4
			
O1—S1—C1—C2	−148.3 (2)	S1—C1—C6—C5	−179.8 (2)
O3—S1—C1—C2	−27.4 (3)	C4—C5—C6—C1	−0.8 (5)
O2—S1—C1—C2	91.5 (2)	C4—N1—C7—C8	−179.3 (3)
O1—S1—C1—C6	32.8 (3)	N1—C7—C8—C9	−4.2 (4)
O3—S1—C1—C6	153.7 (2)	N1—C7—C8—C13	176.4 (3)
O2—S1—C1—C6	−87.4 (2)	C13—C8—C9—O4	−180.0 (3)
C6—C1—C2—C3	−1.0 (5)	C7—C8—C9—O4	0.6 (4)
S1—C1—C2—C3	−179.9 (3)	C13—C8—C9—C10	−0.7 (4)
C1—C2—C3—C4	0.2 (5)	C7—C8—C9—C10	179.9 (3)
C2—C3—C4—C5	0.3 (5)	O4—C9—C10—C11	179.7 (3)
C2—C3—C4—N1	−179.3 (3)	C8—C9—C10—C11	0.5 (5)
C7—N1—C4—C5	−172.3 (3)	C9—C10—C11—C12	0.3 (5)
C7—N1—C4—C3	7.3 (5)	C10—C11—C12—C13	−0.8 (5)
C3—C4—C5—C6	0.0 (5)	C11—C12—C13—C8	0.5 (5)
N1—C4—C5—C6	179.6 (3)	C9—C8—C13—C12	0.2 (4)
C2—C1—C6—C5	1.3 (4)	C7—C8—C13—C12	179.6 (3)

Symmetry codes: (i) −*x*+1, −*y*+1, −*z*+1.

### Hydrogen-bond geometry (Å, °)

**Table:**

*D*—H···*A*	*D*—H	H···*A*	*D*···*A*	*D*—H···*A*
O4—H4···N1	0.82	1.89	2.611 (3)	147
O5—H5A···O1^ii^	0.85	2.07	2.909 (3)	170
O5—H5B···O8^iii^	0.85	1.92	2.750 (3)	167
O6—H6A···O8^i^	0.85	1.93	2.778 (3)	177
O6—H6B···O2^iv^	0.85	1.97	2.802 (3)	166
O7—H7A···O1	0.85	1.95	2.793 (3)	174
O7—H7B···O3^ii^	0.85	1.91	2.757 (3)	172
O8—H8A···O2	0.85	1.90	2.750 (3)	176
O8—H8B···O3^ii^	0.85	2.00	2.851 (3)	176

Symmetry codes: (ii) *x*, *y*−1, *z*; (iii) −*x*+1, *y*−1/2, −*z*+3/2; (i) −*x*+1, −*y*+1, −*z*+1; (iv) *x*, −*y*+3/2, *z*−1/2.
